# Minimal oscillating subnetwork in the Huang-Ferrell model of the MAPK cascade

**DOI:** 10.1371/journal.pone.0178457

**Published:** 2017-06-21

**Authors:** Otto Hadač, František Muzika, Vladislav Nevoral, Michal Přibyl, Igor Schreiber

**Affiliations:** Department of Chemical Engineering, University of Chemistry and Technology, Prague, Czech Republic; SUNY Downstate MC, UNITED STATES

## Abstract

Prompted by the recent growing evidence of oscillatory behavior involving MAPK cascades we present a systematic approach of analyzing models and elucidating the nature of biochemical oscillations based on reaction network theory. In particular, we formulate a minimal biochemically consistent mass action subnetwork of the Huang-Ferrell model of the MAPK signalling that provides an oscillatory response when a parameter controlling the activation of the top-tier kinase is varied. Such dynamics are either intertwined with or separated from the earlier found bistable/hysteretic behavior in this model. Using the theory of stability of stoichiometric networks, we reduce the original MAPK model, convert kinetic to convex parameters and examine those properties of the minimal subnetwork that underlie the oscillatory dynamics. We also use the methods of classification of chemical oscillatory networks to explain the rhythmic behavior in physicochemical terms, i.e., we identify of the role of individual biochemical species in positive and negative feedback loops and describe their coordinated action leading to oscillations. Our approach provides an insight into dynamics without the necessity of knowing rate coefficients and thus is useful prior the statistical evaluation of parameters.

## Introduction

Mitogen-activated protein kinase (MAPK) cascades represent a key step of chemical signal transduction in cellular systems and are widely conserved in eukaryotes [1]. MAPK cascades usually consists of three phosphorylation/dephosphorylation tiers or stages [2]. For example, affinity reaction of tyrosin kinase membrane-bound receptors with extracellular ligands leads to a sequence of reaction steps resulting in the activation of specific intracellular proteins such as Ras. The Ras protein starts the MAPK cascade via activation of a kinase-kinase-kinase enzyme (MAPKKK). This protein then phosphorylates twice the kinase-kinase enzyme (MAPKK) yielding its MAPKKp and MAPKKpp forms in the middle tier. Because the dissociation rate of monophosphorylated enzyme complex MAPKKK-MAPKKp is much faster than the second phosphorylation step yielding MAPKKpp, the double phosphorylation process obeys the distributive enzyme kinetics requiring reassociation of MAPKKp [3]. MAPKKpp finally double phosphorylates the kinase enzyme (MAPK) in the last tier. The single and biphosporylated enzyme forms are deactivated by phospahatases. The biphosphorylated kinase (MAPKpp) then targets different molecules such as transcription factors or cytosolic proteins. The protein modification can lead to cell proliferation, adaption to surrounding environment, cell differentiation, tissue morphogenesis, cell apoptosis and other processes [4] that are tightly connected to autocrine/paracrine communication mediated by membrane receptors and their ligands [5, 6]. MAPK cascades are responsible for signal amplification and ultrasensitivity at certain range of input signal [7]. The sensitivity of MAPK cascades can be characterized by a high value of the Hill cooperative kinetics that suggests switch-like response of the cascade. For example, the Huang-Ferrell model of three-stage MAPK cascade predicts the effective Hill coefficient greater than five [2].

MAPK cascades are involved in various positive and negative feedback loops regulating many intracellular processes [7, 8]. MAPK cascades do not form any purely autonomous reaction network. They are interconnected with other subnetworks such as phosphatidylinositol 3-kinase (PI3K) pathways [9] or are affected by complex signal inputs such as calcium oscillations [10]. However, detailed analyses of MAPK cascades detouched from other processes revealed extraordinary rich behavior so that consideration of cross-talks with other reaction networks or cascades somewhat obscures understanding of the origin of strongly nonlinear dynamical characteristics of MAPK cascades. Consequently, considerable attention has been paid to dynamics of an isolated MAPK cascade or its subsystems.

A large body of work has focused on model formulation and subsequent numerical analysis. Kholodenko [8] studied a three-stage MAPK cascade with MAPKpp inhibiting MAPKKK activity. It was shown that this negative feedback loop can lead to sustained oscillations of kinase activity with the period ranging from minutes to hours. Chickarmane et al. [11] suggested that MAPK networks exhibiting oscillations have to contain at least one double phosphorylation tier with additional negative feedback. However, grater number of tiers produces sustained oscillations more effectively due to the delay between a signal input and negative feedback. Further, Blüthgen et al. [12] found that substrate sequestration due to binding to a kinase or phosphatase can suppress occurrence of oscillations in kinase cascades with simple negative feedback. Nguyen [13] considered a hypothetical arrangement of MAPK cascade with two negative feedbacks in which MAPKpp inhibits the formation of phosphorylated forms of MAPKKK and MAPKK. It was shown that the outer negative feedback (MAPKKK inhibition) promotes oscillatory behavior, whereas the inner suppresses (MAPKK inhibition) the oscillations. Mai and Liu [14] developed a generic mathematical model of three tier MAPK cascade with positive and negative feedbacks and discussed the conditions leading to oscillations, bistability, ultrasensitivity, and transient activation. It was revealed that MAPK cascades exhibit bistable and oscillatory behavior even if no direct positive or negative feedback between MAPKpp and MAPKKK is present. Markevich et al. [15] found that bistable behavior is provided by the distributive enzyme kinetics of double phosphorylation/dephosphorylation in a single tier of MAPK cascades. In such arrangement, the MAPKK and MAPKKp forms of substrate compete for binding sites of MAPKKK, and MAPKKp and MAPKKpp compete for binding sites of phosphatase. Two distinct stable steady states are formed, one rich in in the MAPKK and the other one rich in the MAPKKpp. Kholodenko and Birtwistle in their review [4] summarized the necessary conditions for bistability occurrence: competitive inhibition of a kinase or phosphatase, saturation of respective enzyme by its substrates, and the ratio of phosphorylation/dephosphorylation rate constants in the first phosphorylation must be less than that in the second step. When synthesis and degradation of particular proteins are considered in addition to the enzyme processes in single tier double phosphorylation cascade, sustained oscillations of MAPK activity were observed by Wang et al. [16]. The period of oscillations was determined by protein degradation rate. Occurrence of autonomous oscillations in two-stage or three-stage versions of the Huang-Ferrell model of the MAPK cascade with no extra feedback was reported by Qiao et al. [17] They found large regions of bistabilities and oscillations in the high-dimensional parameter space of the Huang-Ferrell model. Statistical treatment of obtained results confirmed a double phosphorylation subsystem as the necessary condition for emergence of bistability. In agreement with the findings of Wang et al. [16], even temporary consumption or production of a kinase (e.g., represented by the first tier of MAPK cascade) together with the double phosphorylation/deposphorylation motif lead to oscillations.

Another direction in the research of the MAPK cascades is based on purely analytical approach. Conradi et al. [18] found analytical expressions for the location of regions with steady state multiplicities in the parameter space for one tier MAPK cascade. They utilized the reaction network theory developed by Horn and Feinberg [19, 20]. Perez-Millan and Turjanski [21] found that three tier MAPK cascades without extra feedback exhibit so called toric steady states, which allowed to find values of rate constants that correspond to bistability. Ventura et al. [22] approximated each phosphorylation/dephosphorylation step by single variable and pointed out that each such step affects not only downstream members of kinase cascades but also preceding reactions leading to pseudonegative feedback and strongly nonlinear responses to simple stimulus. This method allowed for identification of bistabilities and oscillations in MAPK cascades [23]. A stability based criterion for different biochemical reaction networks including MAPK cascades was introduced by Arcak and Sonntag [24]. Zumsande and Gross [25] used bifurcation analysis to reveal dynamical characteristics of MAPK cascades. Their approach is based on direct parametrization of the Jacobi matrix instead of the right hand sides of governing equations. In addition to bistability and oscillatory behavior, the authors identified quasiperiodic and chaotic dynamics close to a double Hopf bifurcation point in three-tier MAPK cascade. Other techniques of analyzing dynamics of MAPK networks have been used, e.g., Vera et al. [26] used power-law models with possible noninteger and negative reaction orders. Spatial distributions of different forms of kinases between the nucleus and cytosolic membrane were studied by Zhao et al. [27]. They observed traveling waves of kinase concentration originating from the bistable and oscillatory character of the MAPK cascades. Nomura and Okada-Hatakeyama [28] carried out phase response analysis of MAPK cascade and found response functions with negative values of phase shift as well as synchronous and asynchronous oscillations of two coupled MAPK cascades.

Last but not least, there is a growing experimental evidence for oscillatory dynamics in MAPK cascades observed *in vivo*. Sustained oscillations in MAPK phosphorylation were experimentally observed in yeasts continuously exposed to a mating-pheromone stimulus by Hilioti et al. [29]. They found that oscillations in the MAPK cascade formed by Ste11, Ste7, and Fus3 kinases led to periodic mating-gene expression with period of a few hundred minutes. Shankaran et al. [30] reported on sustained oscillations in MAPK cascade in human epithelial cells stimulated by an epidermal growth factor (EGF). Oscillations were persistent for more than 45 cycles with the period of about 15 minutes. Upon exposition of a fibroblast cell lines to fibroblast growth factor stimulus, oscillatory activation of MAPK cascade with the period of about two or three hours were reported by Nakayama et al. [31]. Recently, Hu at al. [32] observed oscillations in MAPK and PI3K signaling cascades upon stimulation of breast cancer cells by EGF. A typical period of observed oscillations was about 20 minutes. The experimental observations suggest that oscillatory dynamics in MAPK cascades may play an important role in the regulation and timing of cell processes. Thus it is increasingly important to fully understand the origin of this behavior.

As suggested in earlier work [4, 11, 15] the MAPK dual-phosphorylation subsystem described in terms of Michaelis-Menten kinetic terms requires competitive inhibition of MAPKKK by MAPKK and MAPKKp that lead to bistable dynamics. Recently we reported on a mass action reaction subnetwork possessing the double phosphorylation motif found in an isolated second or third stage of the MAPK cascade that displays competitive autocatalysis as a major contributor to bistability [33]. With the use of techniques of stoichiometric network analysis established by Clarke [34], we systematically reduced complexity of this motif and obtained a minimal network that still retains bistability. Convex parametrization of the minimal subsystem allowed to obtain analytical formulas for the bistability region in the parameter space. Here we apply this approach to identify a minimal network exhibiting periodic oscillation.

## Methods

### Stoichiometric network analysis (SNA)

The methods of the SNA [34] start with a decomposition of the reaction network into irreducible subsystems called *elementary* or *extreme subnetworks*, subsequently examines linear stability of admissible steady states in each subnetwork and, finally, draws general conclusions on the stability of the network as a whole.

A *reaction network* (also called a *chemical* or *stoichiometric network*) is constructed from a set of *n* chemical species occurring in a set of *r* chemical reactions with given kinetics. A chemical reaction R_*j*_ is specified by providing left and right stoichiometric coefficients $\nu_{ij}^{L},\;\nu_{ij}^{R}$ resp., of species *i* in reaction *j* and the reaction rate *v*_*j*_ as a function of the concentrations *x*_*i*_ of reactants.

Mass balance for each species implies that in a spatially homogeneous (open or closed) isothermal system dynamics is governed by the *evolution equations*, put in a compact form

$$\begin{array}{c} \frac{\text{d}x}{\text{d}t}=N\;v\left(x\right)\;, \end{array}$$ (1)

where $N=\{\nu_{ij}^{R}-\nu_{ij}^{L}\}$ is the stoichiometric matrix; the concentration vector **x** = (*x*_1_, ⋯, *x*_*n*_) has positive components and the reaction rate vector **v** = (*v*_1_, ⋯, *v*_*r*_) has nonnegative components. The concentration dependence of reaction rates is assumed to have a power law form $v=\{v_{j}=k_{j}\prod_{i}x_{i}^{\kappa_{ij}}\}$ where the rate coefficients can be arranged in a vector **k** = (*k*_1_, ⋯, *k*_*r*_) and reaction orders in a matrix **K** = {*κ*_*ij*_}. Inflows and outflows are treated as pseudoreactions. A reaction order of species *i* in reaction *R*_*j*_ can be generally any real number, but for mass action kinetics considered here it is assumed equal to the stoichiometric coefficient of the reactant, $\kappa_{ij}=\nu_{ij}^{L}$. There may be conservation constraints relating certain groups of species that do not flow in or out of the system (such as various enzyme forms within cytosol). The inflow/outflow rates, the rate coefficients and the total concentrations of species subject to conservation constraints represent the parameter space of Eq (1).

At steady state $x^{0}=\left(x_{1}^{0},\cdots ,x_{n}^{0}\right)$ the reaction rate vector **v**^0^ = **v**(**x**^0^) of the network satisfies

$$\begin{array}{c} N\;v^{0}=0\;. \end{array}$$ (2)

The decomposition into elementary subnetworks amounts to finding a set of irreducible solutions of Eq (2) represented by non-negative extreme vectors forming edges of a convex cone in the right null space of **N** of dimension *d* = *r* − rank(**N**). Each of these edges/subnetworks represent a distinct connected pathway encompassing a subset of species. The number *f* of elementary subnetworks may exceed the dimension *d* of the cone. An alternative to the term *elementary subnetwork* is the term *extreme current* used to emphasize a broad analogy to electrical circuits. It is convenient to normalize each elementary subnetwork so that the sum of all reaction rates is equal to one and arrange them as columns in an (*r* × *f*) matrix **E** that can be computed by linear programming algorithms [35] or pathway oriented algorithms [36, 37]. Any non-negative linear combination of the columns of **E** forms a feasible solution of Eq (2),

$$\begin{array}{c} E\;\alpha =v^{0},\;\alpha =\left(\alpha_{1}\geq 0,\cdots ,\alpha_{f}\geq 0\right). \end{array}$$ (3)

Thus any steady state reaction (sub)network can be expressed as a combination of elementary subnetworks.

A useful representation of a (sub)network is provided by a network diagram, which connects species via multi-head, multi-tail arrows representing reactions. The left stoichiometric coefficient $\nu_{ij}^{\text{L}}$ of a species in a reaction is given by the number of ‘feathers’ at the tail, left ‘feathers’ indicate the order *κ*_*ij*_ (for simplicity there is just a straight feather when $\nu_{ij}^{\text{L}}=\kappa_{ij}=1$) and the right coefficient $\nu_{ij}^{\text{R}}$ of a species is indicated by the number of barbs at the head of a reaction arrow.

Identification of elementary subnetworks is useful when examining the stability of the (sub)network at steady state **x**^0^ via eigenvalues of the Jacobi matrix **J** of Eq (1) linearized about the steady state. Of particular interest is emergence of an oscillatory instability when some of the parameters are varied. This occurs when a pair of complex eigenvalues crosses the imaginary axis (a Hopf bifurcation). Also of interest is the saddle-node bifurcation, when a real eigenvalue crosses zero. While the latter has been the subject of our previous work, here we focus on the former.

One of the most convenient features of the SNA approach is that when assessing stability of a steady state network, knowledge of steady state concentrations **x**^0^ can be partly circumvented by switching from the original inflow-kinetic-constraint parameters to convex parameters: the vector of reciprocal steady states, $h=(h_{1}=1/x_{1}^{0},\cdots ,h_{n}=1/x_{n}^{0})$, and the vector of the coefficients ***α***. Then the Jacobi matrix is expressed as [34],

$$\begin{array}{c} J\left(h,\alpha \right)=N\;\text{diag}\left(E\;\alpha \right)\;K^{\text{T}}\;\text{diag}\left(h\right)=-B\;\text{diag}\left(h\right)\;, \end{array}$$ (4)

A preliminary stability analysis can be done by examining the matrix **B**, which does not depend on steady state concentrations. The stability of the subnetwork **v**^0^ = **E** ***α*** is indicated by principal subdeterminants/minors *β*_*ℓ*_ of order *ℓ* = 1, …, *n* of **B**. There are $\left(\begin{array}{c} n\\ \ell \end{array}\right)$ different *β*_*ℓ*_s related to combinations of *ℓ* species out of *n*. If there is a negative *β*_*ℓ*_, then an eigenvalue of **J** has positive real part(s) provided that the values of the reciprocal steady state concentrations of the corresponding *ℓ* species are sufficiently large [34]. At this point there is no clear distinction between a saddle-node and a Hopf bifurcation and a more detailed examination involving **h** must be made, e.g. employing the Routh-Hurwitz criterion [38]. However, a negative principal minor *β*_*ℓ*_ has direct physical interpretation: it implies that positive feedback dominates over negative feedback involving the relevant *ℓ* species. Moreover, when a subnetwork is indicated as unstable, the entire network will display the same type of instability provided that the unstable subnetwork is dominant. This observation makes it possible to introduce a classification of reaction networks which display either steady state bistability or oscillations.

### Classification and role of species in oscillations

A strictly rigorous approach showing that there is an oscillatory instability via Hopf bifurcation is rather technical [38, 39] and does not necessarily provide a physical insight. For that purpose a theory of classification of chemical oscillators has been successively built [40, 41] based partly on rigorous results from the network analysis and partly on heuristics accumulated in the process of analyzing various chemical oscillators ranging from inorganic to biochemical. According to this approach, there are species essential and nonessential for oscillations, the former must be present in the mechanism as dynamical variables, whereas the latter can be kept fixed without losing oscillatory dynamics. Three basic types of essential species that need to be properly embedded in the network’s topology to obtain oscillations in mass action networks having first order autocatalytic step(s) and thereby reflecting majority of realistic mechanisms are: type X (autocatalytic species), type Y (exit or direct inhibition species) and type Z (negative feedback or indirect inhibition species). A negative principal minor *β*_*ℓ*_ of **B** implies positive feedback. Moreover, species that are labeled by the index sequence of that minor are all either type X or type Y species [42]. The minimal subnetwork that involves all the species indicated by *β*_*ℓ*_ may also involve a type Z species that provides for a specific negative feedback so that the subnetwork alone is oscillatory. Alternatively, type Z species and its negative feedback may be present in a combination of the unstable subnetwork with others. Typically, such minimal subnetworks are either directly edges or low-dimensional faces of the convex cone, therefore it is important to know the hierarchy of all *k*-dimensional faces of the cone, *k* = 1, ⋯, *d* − 1. Thus both rigorous mathematical analysis and heuristic physical interpretation in terms of classification of species and explaining their role can be combined to uncover the nature of (bio)chemical oscillators.

## Results

### Reduction to a minimal oscillatory model

**Fig 1:**
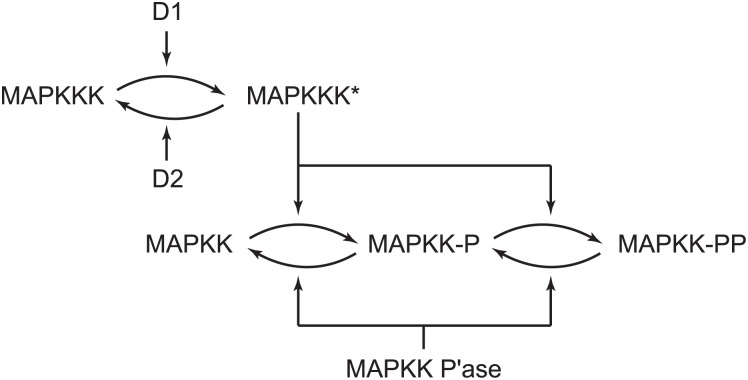
Schematic of the Stages 1 and 2 of the Huang-Ferrell model of the MAPK cascade.

In our earlier work we started with applying mass action kinetics to express each Michaelian step in the Stage 2 of the Huang-Ferrell model of MAPK cascade and reduced it to a minimal biochemically consistent model possessing bistability based on competitive autocatalysis [33]. When mass action kinetics are applied, systematic simulations of Qiao *et al.* [17] have indicated that Stages 1 and 2 should be involved in oscillations, see Fig 1. For convenience, we use abbreviated notation for various enzymes in Fig 1 summarized in Table 1. In the first stage activation of the kinase A using an enzyme D_1_ to A* and simultaneous deactivation via another enzyme D_2_ takes place while the subsequent Stage 2 involves a sequential double phosporylation/double dephosphorylation in a scheme known as distributive model. Specifically, the activated kinase A* phosphorylates two substrates, B and its phosphorylated form B_1_; the double phosphorylated form B_2_ as well as the single phosphorylated form B_1_ are dephosphorylated by the phosphatase C.

**Table 1:** Notation used for chemical species.

Symbol	Chemical species
A	MAPKKK
A*	MAPKKK*
B	MAPKK
B_1_	MAPKKP
B_2_	MAPKKPP
C	MAPKKP’ase
A*B	MAPKKK*MAPKK
A*B_1_	MAPKKK*MAPKKP
CB_1_	MAPKKP’aseMAPKKP
CB_2_	MAPKKP’aseMAPKKPP
D_1_	enzyme activating MAPKKK
D_2_	enzyme deactivating MAPKKK

Using the approach outlined in the section Methods, we take the two-stage subset of the MAPK cascade and reduce it to a minimal oscillatory network and simultaneously show how the minimal model for bistability fits within the oscillatory network. Provided that each of the enzymatic reactions obeys Michaelis-Menten reaction steps, the Stages 1 and 2 involve *n* = 14 chemical species and *r* = 18 reactions (forward and reverse steps are counted as separate) summarized in Table 2 that follow mass action kinetics.

**Table 2:** The reaction mechanism of the MAPK Stage 1 + 2.

No.	Reaction	Reaction rate
(1)	$\text{B}+{\text{A}}^{*}⇌{\text{A}}^{*}\text{B}$	$v_{1}=k_{1}\left[{\text{A}}^{*}\right]\left[\text{B}\right]-k_{\text{-}1}\left[{\text{A}}^{*}\text{B}\right]$
(2)	A*B → A* + B_1_	*v*_2_ = *k*_2_[A*B]
(3a)	${\text{B}}_{1}+{\text{A}}^{*}⇌{\text{A}}^{*}{\text{B}}_{1}$	$v_{3a}=k_{3a}\left[{\text{A}}^{*}\right]\left[{\text{B}}_{1}\right]-k_{\text{-}3a}\left[{\text{A}}^{*}{\text{B}}_{1}\right]$
(3b)	A*B_1_ → B_2_ + A*	*v*_3*b*_ = *k*_3*b*_[A*B_1_]
(4a)	${\text{B}}_{2}+\text{C}⇌{\text{CB}}_{2}$	$v_{4a}=k_{4a}\left[\text{C}\right]\left[{\text{B}}_{2}\right]-k_{\text{-}4a}\left[{\text{CB}}_{2}\right]$
(4b)	CB_2_ → B_1_ + C	*v*_4*b*_ = *k*_4*b*_[CB_2_]
(5a)	${\text{B}}_{1}+\text{C}⇌{\text{CB}}_{1}$	$v_{5a}=k_{5a}\left[\text{C}\right]\left[{\text{B}}_{1}\right]-k_{\text{-}5a}\left[{\text{CB}}_{1}\right]$
(5b)	CB_1_ → B + C	*v*_5*b*_ = *k*_5*b*_[CB_1_]
(6a)	${\text{D}}_{1}+\text{A}⇌{\text{D}}_{1}\text{A}$	$v_{6a}=k_{6a}\left[\text{A}\right]\left[{\text{D}}_{1}\right]-k_{\text{-}6a}\left[{\text{D}}_{1}\text{A}\right]$
(6b)	D_1_A → D_1_ + A*	*v*_6*b*_ = *k*_6*b*_[D_1_A]
(7a)	${\text{D}}_{2}+{\text{A}}^{*}⇌{\text{D}}_{2}{\text{A}}^{*}$	$v_{7a}=k_{7a}\left[{\text{A}}^{*}\right]\left[{\text{D}}_{2}\right]-k_{\text{-}7a}\left[{\text{D}}_{2}{\text{A}}^{*}\right]$
(7b)	D_2_A* → D_2_ + A	*v*_7*b*_ = *k*_7*b*_[D_2_A*]

We assume spatial homogeneity of the reaction environment to cast the model in terms of ordinary differential equations. This is certainly a simplification, because despite small size of the cell, diffusive transport within cytosol may be effectively hindered due to the presence of endoplasmic reticulum and other subcellular structures with complex geometry. A simple calculation of diffusion time scale in a cell of a characteristic size 1 × 10^−5^*m* with a diffusing protein having effective diffusivity 1 × 10^−11^ m^2^s^−1^ leads to a diffusion time 10 s, which is much shorter than periods observed in experiments. Thus the oscillatory dynamics will be primarily determined by kinetics and on the qualitative level of description, the assumption of spatial homogeneity is relevant. The time evolution of the concentrations of the species involved in Stages 1 and 2 is given by the evolution equations (see also Eq (1)):

$$\begin{array}{c} \frac{\text{d}x}{\text{d}t}=N\;v\left(x\right)\;, \end{array}$$ (5)

with the stiochiometric matrix

$$\begin{array}{c} N=[\begin{array}{cccccccccccc} 0 & 0 & 0 & 0 & 0 & 0 & 0 & 0 & -1 & 1 & 0 & 0\\ 0 & 0 & 0 & 0 & 0 & 0 & 0 & 0 & 0 & 0 & -1 & 1\\ 0 & 0 & 0 & 0 & 0 & 0 & 0 & 0 & 1 & -1 & 0 & 0\\ 0 & 0 & 0 & 0 & 0 & 0 & 0 & 0 & 0 & 0 & 1 & -1\\ 0 & 0 & 0 & 0 & 0 & 0 & 0 & 0 & -1 & 0 & 0 & 1\\ -1 & 1 & -1 & 1 & 0 & 0 & 0 & 0 & 0 & 1 & -1 & 0\\ -1 & 0 & 0 & 0 & 0 & 0 & 0 & 1 & 0 & 0 & 0 & 0\\ 0 & 1 & -1 & 0 & 0 & 1 & -1 & 0 & 0 & 0 & 0 & 0\\ 0 & 0 & 0 & 1 & -1 & 0 & 0 & 0 & 0 & 0 & 0 & 0\\ 0 & 0 & 0 & 0 & -1 & 1 & -1 & 1 & 0 & 0 & 0 & 0\\ 1 & -1 & 0 & 0 & 0 & 0 & 0 & 0 & 0 & 0 & 0 & 0\\ 0 & 0 & 1 & -1 & 0 & 0 & 0 & 0 & 0 & 0 & 0 & 0\\ 0 & 0 & 0 & 0 & 0 & 0 & 1 & -1 & 0 & 0 & 0 & 0\\ 0 & 0 & 0 & 0 & 1 & -1 & 0 & 0 & 0 & 0 & 0 & 0 \end{array}], \end{array}$$ (6)

and rate expressions as in Table 2. The ordering of species (rows) is D_1_, D_2_, D_1_A, D_2_A*, A, A*, B, B_1_, B_2_, C, A*B, A*B_1_, CB_1_, CB_2_ and that of reactions (columns) as in Table 2. Concentrations of other species (ATP, water) are assumed fixed due to elevated concentrations (also referred to as pool condition) and included in the rate coefficients *k*_*j*_. Since the total amounts of all five enzymes are conserved within the system, Eq (5) must obey the conservation constraints

$$\begin{array}{c} {\text{A}}_{\text{tot}}=\left[\text{A}\right]+\left[{\text{A}}^{*}\right]+\left[{\text{A}}^{*}\text{B}\right]+\left[{\text{A}}^{*}{\text{B}}_{1}\right]+\left[{\text{D}}_{1}\text{A}\right]+\left[{\text{D}}_{2}{\text{A}}^{*}\right]\;, \end{array}$$ (7)

$$\begin{array}{c} {\text{B}}_{\text{tot}}=\left[\text{B}\right]+\left[{\text{B}}_{1}\right]+\left[{\text{B}}_{2}\right]+\left[{\text{A}}^{*}\text{B}\right]+\left[{\text{A}}^{*}{\text{B}}_{1}\right]+\left[{\text{CB}}_{1}\right]+\left[{\text{CB}}_{2}\right]\;, \end{array}$$ (8)

$$\begin{array}{c} {\text{C}}_{\text{tot}}=\left[\text{C}\right]+\left[{\text{CB}}_{1}\right]+\left[{\text{CB}}_{2}\right]\;, \end{array}$$ (9)

$$\begin{array}{c} {\text{E}}_{\text{1,tot}}=\left[{\text{D}}_{1}\right]+\left[{\text{D}}_{1}\text{A}\right]\; \end{array}$$ (10)

$$\begin{array}{c} {\text{E}}_{\text{2,tot}}=\left[{\text{D}}_{2}\right]+\left[{\text{D}}_{2}{\text{A}}^{*}\right]\;. \end{array}$$ (11)

**Fig 2:**
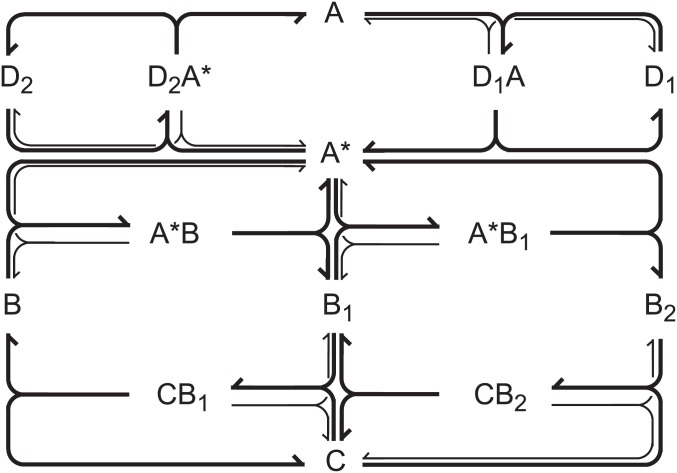
Network diagram of the two-stage model of the MAPK cascade. The oscillatory subnetwork is indicated by thick lines.

This model has effectively rank(**N**) = 14 − 5 = 9 independent variables since each concentration constraint makes one of the respective enzyme forms dependent on others. The corresponding network diagram is shown in Fig 2. The decomposition into elementary subnetworks and stability analysis applied directly to the reaction network defined by Eq (5) indicates *f* = 9 elementary subnetworks. Since the dimension of the steady state cone is *d* = *r* − rank(**N**) = 18 − 9 = 9 and *f* = *d*, the cone is simplicial, i.e., it is generated by a minimal number of edges. Six elementary subnetworks are simple forward-reverse pairs corresponding to reversible enzyme-substrate complex formation. Unsurprisingly, these subnetworks can never give rise to an instability when considered separately or mutually mixed. The remaining three subnetworks do not include any reverse steps; the first one involves phosphorylation of B combined with dephosphorylation of B_1_, the second involves phosphorylation of B_1_ together with dephosphorylation of B_2_, the third one involves activation of A and deactivation of A*. Each of them taken separately is also stable. In fact, the only unstable subnetwork (in the sense of possessing a negative principal minor *β*_*ℓ*_ and therefore a positive matrix feedback, see section Methods) is the combination of first and second phosphorylation, which is exactly the subnetwork found in our earlier work [33] to generate bistability. It is also straightforward to find out that the simplest way of obtaining an oscillatory instability emerging via Hopf bifurcation is to combine all three irreversible elementary subnetworks, in other words, to combine the irreversible Stage 2 possessing positive feedback with the irreversible Stage 1 that provides for suitable negative feedback. This oscillatory subnetwork is indicated in Fig 2 by thick lines. Adding any of the six equilibrium subnetworks to the double-phosphorylation subnetwork dose not provide negative feedback leading to oscillations.

**Fig 3:**
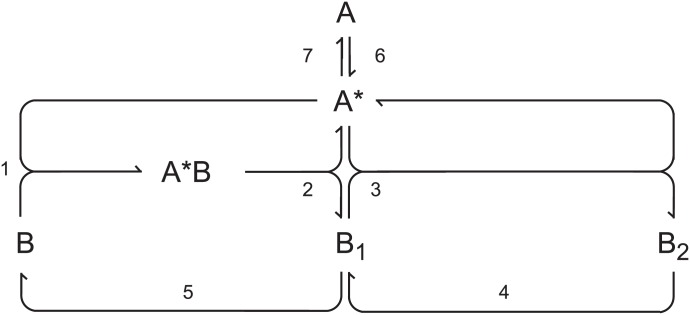
Network diagram of the minimal oscillatory MAPK subnetwork.

However, the irreversible form of the model still possesses 9 effective dynamical variables, which makes further analysis cumbersome. By arguments analogous to those in [33] this scheme can be significantly reduced while keeping its oscillatory properties by merging the steps (3a) and (3b) and assuming that the activation/deactivation enzymes D_1_, D_2_ and the phosphatase C are in pool condition. This implies first order activation/deactivation as well as first order dephosphorylation of B_2_ to B_1_ and B_1_ to B. As a result, we obtain the simplified mechanism found in Table 3 and the corresponding reaction network is shown in Fig 3. The rate coefficients are essentially those of the forward steps in the reversible reactions in the original network with included concentrations of the pool species.

**Table 3:** The minimal oscillatory mechanism of the MAPK cascade.

No.	Reaction	Reaction rate
(1)	B + A* → A*B	*v*_1_ = *k*_1_[A*][B]
(2)	A*B → A* + B_1_	*v*_2_ = *k*_2_[A*B]
(3)	B_1_ + A* → B_2_ + A*	*v*_3_ = *k*_3_[A*][B1]
(4)	B_2_ → B_1_	*v*_4_ = *k*_4_[B_2_]
(5)	B_1_ → B	*v*_5_ = *k*_5_[B_1_]
(6)	A → A*	*v*_6_ = *k*_6_[A]
(7)	A* → A	*v*_7_ = *k*_7_[A*]

Dynamics of the minimal oscillating subnetwork is described by

$$\begin{array}{ccc} \frac{\text{d}x_{1}}{\text{d}t} & = & k_{6}x_{6}-k_{7}x_{1}-k_{1}x_{1}x_{2}+k_{2}x_{4}, \end{array}$$ (12)

$$\begin{array}{ccc} \frac{\text{d}x_{2}}{\text{d}t} & = & k_{5}x_{3}-k_{1}x_{1}x_{2}, \end{array}$$ (13)

$$\begin{array}{ccc} \frac{\text{d}x_{3}}{\text{d}t} & = & k_{2}x_{4}-k_{3}x_{1}x_{3}+k_{4}x_{5}-k_{5}x_{3}, \end{array}$$ (14)

$$\begin{array}{ccc} \frac{\text{d}x_{4}}{\text{d}t} & = & k_{1}x_{1}x_{2}-k_{2}x_{4}, \end{array}$$ (15)

$$\begin{array}{ccc} \frac{\text{d}x_{5}}{\text{d}t} & = & k_{3}x_{1}x_{3}-k_{4}x_{5}, \end{array}$$ (16)

$$\begin{array}{ccc} \frac{\text{d}x_{6}}{\text{d}t} & = & k_{7}x_{1}-k_{6}x_{6}. \end{array}$$ (17)

where *x*_1_, *x*_2_, *x*_3_, *x*_4_, *x*_5_, and *x*_6_ stand for [A*], [B], [B_1_], [A*B], [B_2_], and [A], respectively. There are two conservation equations for various forms of the two types of kinases,

$$\begin{array}{ccc} {\text{A}}_{\text{tot}} & = & x_{1}+x_{4}+x_{6}\;, \end{array}$$ (18)

$$\begin{array}{ccc} {\text{B}}_{\text{tot}} & = & x_{2}+x_{3}+x_{4}+x_{5}\;. \end{array}$$ (19)

Decomposition of the minimal system at steady state yields only three elementary subnetworks, E_1_ includes reactions 6, 7 (activation/deactivation), E_2_ includes reactions 1, 2 and 5 (first phophorylation/second dephosphorylation), E_3_ includes reactions 3 and 4 (second phophorylation/first dephosphorylation). The 2-dimensional face of the cone formed by E_2_ and E_3_ constitutes the minimal bistable system [33] and coupling of E_1_ contributes to negative feedback necessary for oscillations. The steady state cone has very simple structure by being a three-dimensional open simplicial cone. Below we first use numerical analysis [43, 44] to show bifurcation behavior and transitions from bistable to oscillatory dynamics and focus on explaining the nature of oscillations by using the classification of chemical oscillators [40, 41]. Then we show that the steady states can be found analytically in kinetic parametrization and used to partly construct the bifurcation diagram and finally we use stability analysis in convex parameters to prove the presence of a Hopf bifurcation.

### Numerical bifurcation analysis and classification of oscillations

Any steady state reaction vector **v**^0^ = **v**(**x**^**0**^) of Eqs (12)–(17) can be expressed as a non-negative linear combination of E_1_, E_2_ and E_3_ (see Eq (3)):

$$\begin{array}{c} E\alpha =\left[\begin{array}{ccc} 0 & 0 & 1/3\\ 0 & 0 & 1/3\\ 0 & 1/2 & 0\\ 0 & 1/2 & 0\\ 0 & 0 & 1/3\\ 1/2 & 0 & 0\\ 1/2 & 0 & 0 \end{array}\right]\left(\begin{array}{c} \alpha_{1}\\ \alpha_{2}\\ \alpha_{3} \end{array}\right)=\left(\begin{array}{c} k_{1}x_{1}^{0}x_{2}^{0}\\ k_{2}x_{4}^{0}\\ k_{3}x_{1}^{0}x_{3}^{0}\\ k_{4}x_{5}^{0}\\ k_{5}x_{3}^{0}\\ k_{6}x_{6}^{0}\\ k_{7}x_{1}^{0} \end{array}\right)=v\left(x^{0}\right), \end{array}$$ (20)

The stoichiometric network theory indicates that only combination of E_2_ and E_3_ provides an unstable subnetwork, and there is one negative principal minor *β*_3_ corresponding to the species A*, B and B_1_. Those species should have small concentrations relative to others for the steady state to be unstable. Guided by this analysis, for numerical calculations we can arbitrarily choose *α*_2_ = *α*_3_ = 1 and $x_{1}^{0}=x_{2}^{0}=x_{3}^{0}=0.1\;\text{nM},\;x_{4}^{0}=x_{5}^{0}=1\;\text{nM}$. Eq (20) implies that the values of rate coefficients of the first five reactions are *k*_1_ = 1/3 × 10^2^ nM^−1^s^−1^, *k*_2_ = 1/3 s^−1^, *k*_3_ = 50 nM^−1^s^−1^, *k*_4_ = 1/2 s^−1^, *k*_5_ = 1/3 × 10^1^ s^−1^ and from Eq (19) we obtain B_tot_ = 2.2 nM. We assume these parameters to be fixed. Depending on the type of parametrization, we choose a pair of free parameters: i) for the kinetic parametrization we additionally fix *k*_7_ at 0.7 s^−1^ while A_tot_ and *k*_6_ are allowed to vary, ii) for the convex parametrization *α*_1_ and $x_{6}^{0}$ are the free parameters. The choice of *k*_6_ is motivated by step 6 being the activation of the top tier enzyme MAPKKK, which is initialized by an externally controlled ligand while A_tot_ specifies the total amount of MAPKKK available. The choice of free convex parameters is complementary, *α*_1_ controls the coupling of inactive MAPKKK to the unstable subnetwork and $x_{6}^{0}$ is the amount of inactive MAPKKK available. Kinetic parameters are used in numerical calculations and also to analytically express steady states, while convex parameters are useful for analysis proving the presence of oscillations and, in addition, provide for guidance and insight.

In terms of convex parameters, it is convenient to view the system’s steady state as a mixture *α*_1_E_1_ + E_2_ + E_3_. The steady state is stable when *α*_1_ is large enough due to strong negative feedback holding the unstable subnetwork back, but a Hopf bifurcation occurs as *α*_1_ is decreased and the unstable subnetwork becomes dominant, resulting in emergence of oscillations. Eventually, when *α*_1_ approaches zero oscillations are lost.

**Fig 4:**
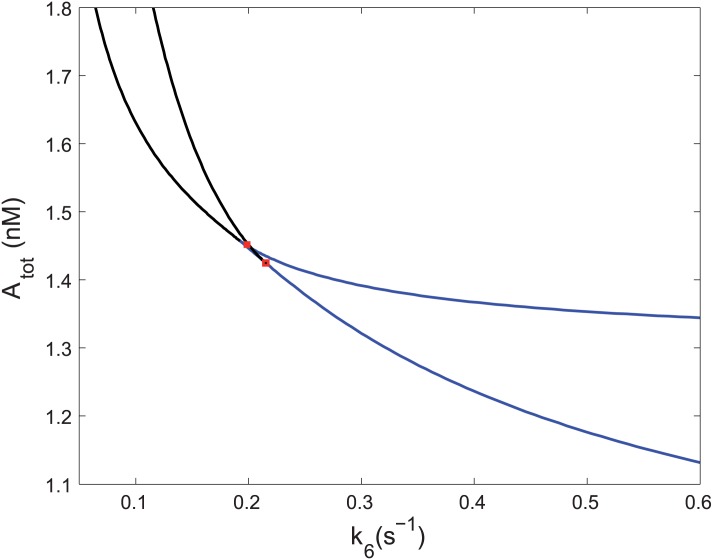
Bifurcation diagram in the parameter plane A_tot_ vs *k*_6_. Values of fixed parameters are given in the text. Blue curve—saddle-node bifurcation delimiting region of three steady states, black curve—Hopf bifurcation, red square—Bogdanov-Takens point.

Conditions for oscillatory behavior are indicated by a two-parameter bifurcation diagram in the plane of *k*_6_ and A_tot_ shown in Fig 4. As expected, there is a cusp-shaped region of bistability delimited by two branches of saddle-node bifurcation curves meeting at a cusp point. There is also an adjacent region of oscillations in the upward direction delimited by two curves of Hopf bifurcation making for the well-known cross-shaped phase diagram. The two branches of the Hopf bifurcation terminate at the saddle-node curve via Bogdanov-Takens points located very near the cusp. Oscillations occur mostly within the region delimited by the Hopf curves, although strictly speaking, there is a narrow belt of oscillations coexisting with one stable steady state surrounding the main oscillatory region due to a subcritical Hopf bifurcation [45]. Notably, the oscillatory region is delimited to values of *k*_6_ markedly smaller than the fixed value of *k*_7_ = 0.7 s^−1^ signifying that the activation of A must be slower than the deactivation of A*. This observation points to a more general feature implied by the negative minor *β*_3_ for the unstable subnetwork: occurrence of any instability requires the steady state values of A*, B and B_1_ to be small relative to other species. More specifically, $x_{1}^{0}$ for A* must be less than $x_{6}^{0}$ for A, which by virtue of Eq (20) implies $k_{6}/k_{7}=x_{1}^{0}/x_{6}^{0}<1$. The oscillatory region disappears as *k*_6_ approaches zero. In this limit only unique stable steady state occurs but bistability would be obtained if both *k*_6_ and *k*_7_ were vanishing.

**Fig 5:**
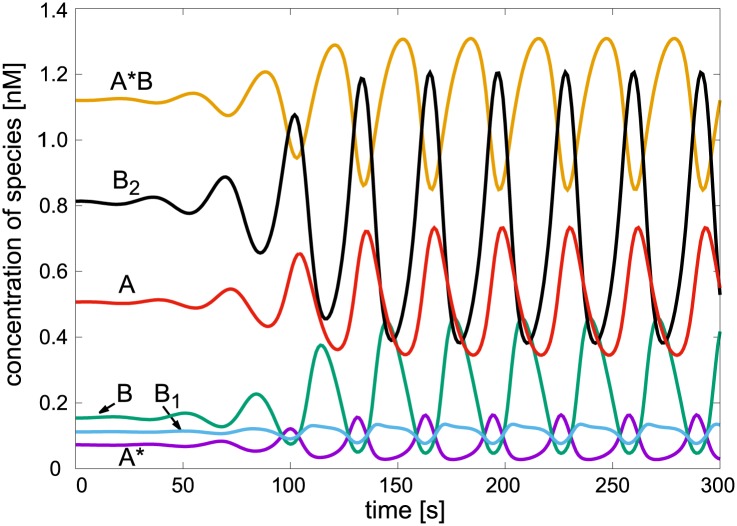
Oscillatory dynamics in the minimal model. Parameters: *k*_6_ = 0.1 s^−1^, A_tot_ = 1.7 nM, other parameters are given in the text.

To analyze the nature of oscillatory dynamics, we choose a sample point within the corresponding region from Fig 4. The oscillatory waveform is shown in Fig 5. Using the classification of chemical oscillators [41, 42] we can determine the role of species played in forming the oscillations (for a brief account see section Methods). Firstly, species essential and nonessential for the oscillations must be distinguished. There are several methods available, but the most straightforward one, applicable when a mechanism given, is to fix concentration of a selected species and find out whether oscillations in the modified network can be recovered. If so, the species is nonessential. Note that fixing a species’ concentration means that the species is buffered or set to a pool condition, nonetheless it is still present in the mechanism.

The species with small concentrations as required by the negative principal minor *β*_3_ (i. e., B, B_1_, A*) are always essential. By applying the fixing method to the other species the resulting classification is as follows: i) A is nonessential and may be assumed in pool condition, as already assumed for D_1_, D_2_ and C, ii) the other two species play a mutually complementary role, if B_2_ is fixed and A*B is kept as dynamical variable, oscillations are recovered and vice versa. Thus both B_2_ and A*B are essential for oscillations but only one of them suffices as dynamical variable at a time.

Next, based on Fig 5 the essential species can be classified by the method of mutual phase shifts. The low-concentration species are arranged in two groups, B and B_1_ are oscillating in-phase, whereas A* is oscillating anti-phase (i.e., shifted by approximately half a cycle). The large-concentration species B_2_ and A*B are mutually anti-phase. At the same time, B_2_ is advancing B and B_1_ but delaying A*, whereas A*B has just the opposite phase shift. These observations suggest that the species B, B_1_ are the autocatalytic species, A* is the exit (direct inhibition, type Y) species and either B_2_ or A*B or both simultaneously play the role of the negative feedback (type Z) species. Importantly, phase shift signatures can be taken as a measure of qualitative agreement between model/mechanism and experiments.

An interesting feature of the MAPK mechanism is that, unlike in many other chemical oscillators [40], there is no cycle that would mutually connect the autocatalytic species B and B_1_ with no other species in the cycle. As already pointed out in our previous work [33], there is a topological feature in the network called competitive autocatalysis that is the central point contributing to positive feedback. Namely, type X species B and B_1_ compete for the type Y species A*. Using the identification of a positive feedback subnetwork and the indentification of essential species, the self-accelerating effect can be explained by carefully examining the phase relations and oscillatory amplitudes of species shown in Fig 5. For the sake of clarity, let us initially assume that parameters are chosen so that activation/deactivation steps 6 and 7 are turned off, the system is in a bistable region and the current steady state is fully phosphorylated, i.e., B and B_1_ are low and B_2_ is high. When the concentration of B is sufficiently increased above its steady state level, A* is consumed by rapid step 1. Because step 2 is much slower, the complex A*B tends to accumulate so that B_1_ and A* form by step 2 only after a delay. The available B_1_ competitively consumes A* via step 3 and is reformed from B_2_ by the slow step 4. However, because concentration of B_2_ is high, the rate of formation of B_1_ is significant. Therefore all three steps 3, 4 and 5 run at a high rate, successively recycling B_1_ and B so that they accumulate at the expense of B_2_ until A* is depleted. In the absence of negative feedback made possible by activation/deactivation steps 6 and 7, this process eventually leads to accumulation of B while both B_1_ and B_2_ maintain low level. Thus a switch from phosphorylated to unphosphorylated steady state occurs. A reverse switch is achieved by adding A*, which triggers the phosphorylation process. Thus positive feedback is present within the group of species B, B_1_ and A* as suggested by the principal minor *β*_3_.

If the steps 6 and 7 are included, then the competitive autocatalysis is controlled by reversible conversion of A to A* and oscillations can occur. Under these conditions there is an alternation of the autocatalytic phase and an inhibitory phase. During the autocatalytic phase B is accumulated and toward the end A* and B_2_ are depleted which brings the autocatalytic growth of B and B_1_ to an end. As a result, the rate of step 6 is higher than that of step 7 and thus activation prevails and A* starts to regenerate. At the same time the complex A*B has become accumulated to the point when the rate of the slow step 2 is significant thereby producing A* and B_1_ which immediately recombine via fast step 3 to regenerate B_2_. During this phase B and B_1_ are being depleted at an increased pace due to continual supply of A*. Therefore the rate of step 2 overcomes that of step 1 and concentration of A*B reaches maximum and begins to decrease. Subsequently, production of the inhibitory species A* ceases as the removal of A* by step 7 becomes faster than its production by step 6. Thus phosphorylation via steps 2 and 3 diminishes while dephosphorylation via steps 4 and 5 is initiated and a new autocatalytic phase starts off.

Negative feedback is achieved by coordinated action of the complex A*B (type Z) with the activated kinase A* (type Y) and is marked by phase delay of A*B with respect to unphosphorylated and mono-phosphorylated kinases B and B_1_ (type X species). As mentioned earlier, even if B_2_ is buffered, the oscillations are preserved. Likewise, if A*B is buffered, the decisive role of negative feedback species is taken over by B_2_. These ultimately simplified suboscillators are a direct consequence of the oscillatory clockwork described above but represent probably too crude an approximation of the original MAPK mechanism.

**Fig 6:**
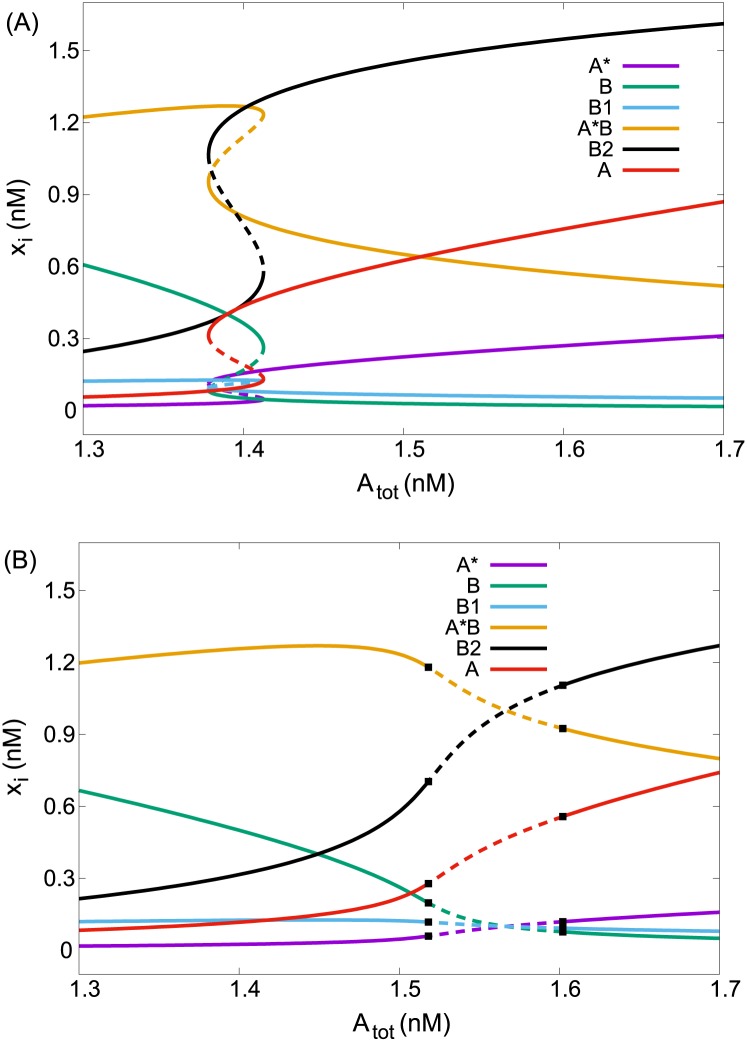
One-parameter bifurcation diagram. Dependence of the steady state values of all six components on A_*tot*_, (A) *k*_6_ = 0.25 s^−1^, (B) *k*_6_ = 0.15 s^−1^. Other parameters are given in text. Full line—stable, dashed line—unstable, black square—Hopf bifurcation.

A complementary view of the role of species in oscillations is provided by looking at the dependence of steady state values on specific parameters near the Hopf bifurcation and in the range of multiple steady states. In Fig 6 we plot the physically meaningful (i.e., nonnegative) steady states in dependence on A_*tot*_ for two fixed values of *k*_6_. The indicative property is the increase/decrease (or upregulation/downregulation) of steady state values of the essential species as A_*tot*_ is increased [40]. The switch in Fig 6A is downwards for A*B, B and B_1_ and upwards for A* and B_2_. Consistently, the corresponding curves in Fig 6B are sloping down/up close to the Hopf bifurcation. The low-concentration species are separated to downregulating (B, B_1_) and upregulating (A*) with respect to A_*tot*_, indicating their opposing role. The key feature in high-concentration species is downregulation of A*. It is in fact a negative self-regulation, which is a distinguishing property of negative feedback (type Z) species [40]. B_2_ is upregulating, i.e., complementary to A*B. However, B_2_ is downregulating with respect to itself (not shown), marking it as the second type Z species. In addition, downregulation with increasing A_*tot*_ of B and B_1_ indicates their autocatalytic role and upregulation of A* its inhibitory role [41]. Thus the concentration shift analysis is leading to the same results as the phase shift analysis.

### Kinetic parametrization

At steady state, Eqs (12)–(17) together with the conservation Eqs (18) and (19) can be solved analytically using software for symbolic manipulations (Matlab, Maple) to yield expressions for steady state concentrations, see Appendix for the corresponding expressions. There are three independent solutions $x_{j}^{\left(i\right)},x_{j}^{\left(ii\right)},x_{j}^{\left(iii\right)},\;j=1,\cdots ,6$. Upon discarding the complex and negative roots, the Eqs (31)–(52) show that there are up to three physically plausible steady states and explicitly specify conditions for the boundary of the region of multiplicity obtained when any two roots merge, i.e., *b*_2_ = 0 or *b*_4_ = *b*_2_ + *b*_3_ or *b*_4_ = *b*_2_ − *b*_3_. An analytical formula expressing conditions for the Hopf bifurcation is obtained by substituting the steady states into the Jacobian matrix of Eqs (12)–(17) and requiring a pair of complex conjugate eigenvalues be pure imaginary. This is more convenient to perform in convex coordinates as shown below. However, an indication of the presence of oscillations is readily obtained by evaluating the Jacobian matrix at the cusp point where all three solutions merge into one, i.e., when *b*_2_ = 0 and *b*_3_ = *b*_4_ and calculating eigenvalues for any chosen set of parameters. There are always three vanishing eigenvalues, two of them because of concentration constraints (Eqs (18) and (19)) and the third one because of the cusp bifurcation point. The remaining eigenvalues are either all negative, or one may become positive. The latter is directly pointing to an adjacent oscillatory region, since when parameters are properly readjusted away from the cusp point, the bifurcating zero eigenvalue becomes positive so that there is a pair of real positive eigenvalues which upon further variation of a parameter become complex conjugate and ultimately pure imaginary, i.e., a Hopf bifurcation is reached.

### Convex parametrization

As outlined in the section Methods, dynamical Eqs (12)–(17) can be reformulated in convex parameters resulting in

$$\begin{array}{ccc} \frac{\text{d}X_{1}}{\text{d}t} & = & \frac{1}{3}\alpha_{3}h_{1}\left(X_{4}-X_{1}X_{2}\right)+\frac{1}{2}\alpha_{1}h_{1}\left(X_{6}-X_{1}\right), \end{array}$$ (21)

$$\begin{array}{ccc} \frac{\text{d}X_{2}}{\text{d}t} & = & \frac{1}{3}\alpha_{3}h_{2}\left(X_{3}-X_{1}X_{2}\right), \end{array}$$ (22)

$$\begin{array}{ccc} \frac{\text{d}X_{3}}{\text{d}t} & = & \frac{1}{3}\alpha_{3}h_{3}\left(X_{4}-X_{3}\right)+\frac{1}{2}\alpha_{2}h_{3}\left(X_{5}-X_{1}X_{3}\right), \end{array}$$ (23)

$$\begin{array}{ccc} \frac{\text{d}X_{4}}{\text{d}t} & = & \frac{1}{3}\alpha_{3}h_{4}\left(X_{1}X_{2}-X_{4}\right), \end{array}$$ (24)

$$\begin{array}{ccc} \frac{\text{d}X_{5}}{\text{d}t} & = & \frac{1}{2}\alpha_{2}h_{5}\left(X_{1}X_{3}-X_{5}\right), \end{array}$$ (25)

$$\begin{array}{ccc} \frac{\text{d}X_{6}}{\text{d}t} & = & \frac{1}{2}\alpha_{1}h_{6}\left(X_{1}-X_{6}\right), \end{array}$$ (26)

where $X_{j}=x_{j}/x_{j}^{0}$ are dimensionless concentrations of respective species scaled with corresponding reference steady state values taken as parameters. For convenience, reciprocal values are used instead, $h_{j}=1/x_{j}^{0}$. Convex parameters are *h*_1_, *h*_2_, *h*_3_, *h*_4_, *h*_5_, *h*_6_ and *α*_1_, *α*_2_, *α*_3_. In order to set definite conservation constraints, A_*tot*_ and B_*tot*_ are assumed to be fixed and the conservation equations are

$$\begin{array}{ccc} {\text{A}}_{\text{tot}} & = & \frac{X_{1}}{h_{1}}+\frac{X_{4}}{h_{4}}+\frac{X_{6}}{h_{6}}\;, \end{array}$$ (27)

$$\begin{array}{ccc} {\text{B}}_{\text{tot}} & = & \frac{X_{2}}{h_{2}}+\frac{X_{3}}{h_{3}}+\frac{X_{4}}{h_{4}}+\frac{X_{5}}{h_{5}}\;. \end{array}$$ (28)

As in the kinetic parametrization, there are up to three non-negative steady states that can be expressed analytically by formulas analogous to Eqs (31)–(38). However, with the aim of determining conditions for emergence of oscillations, it is unnecessary to use all three roots, it suffices to use any of them as a reference solution, since the system is parametrically overdetermined [34] and every steady state of the system can be reached by variation of the convex parameters. By virtue of the reparametrization, one of the solutions of Eqs (27) and (28) is simply *X*_*j*_ = 1, *j* = 1, ⋯, 6. By taking this solution, the Jacobian matrix according to Eq (4) has a form amenable to algebraic manipulations. In particular, Routh-Hurwitz criterion [38] can be applied giving the necessary conditions for a Hopf bifurcation in an analytic form. To this end coefficients of the characteristic polynomial can be expressed in terms of convex parameters and Routh-Hurwitz determinants [38] can be evaluated. The characteristic equation is in the form

$$\begin{array}{c} \lambda^{2}\left(c_{0}\lambda^{4}+c_{1}\lambda^{3}+c_{2}\lambda^{2}+c_{3}\lambda +c_{4}\right)=0\;, \end{array}$$ (29)

where the roots λ_*j*_ are the eigenvalues. Because of the concentration constraints, two roots are zero. When a parameter is varied, a Hopf bifurcation from a stable steady state occurs when a pair of complex conjugate eigenvalues crosses imaginary axis from left to right and the remaining two eigenvalues have negative real parts. This condition is fulfilled when certain inequalities and equalities for the Routh-Hurwitz determinants are met [38], which in our case reads

$$\begin{array}{c} c_{0}>0,\;c_{1}>0,\;c_{1}c_{2}>c_{0}c_{3}\;\text{and}\;c_{3}\left(c_{1}c_{2}-c_{0}c_{3}\right)-c_{1}^{2}c_{4}=0\;. \end{array}$$ (30)

**Fig 7:**
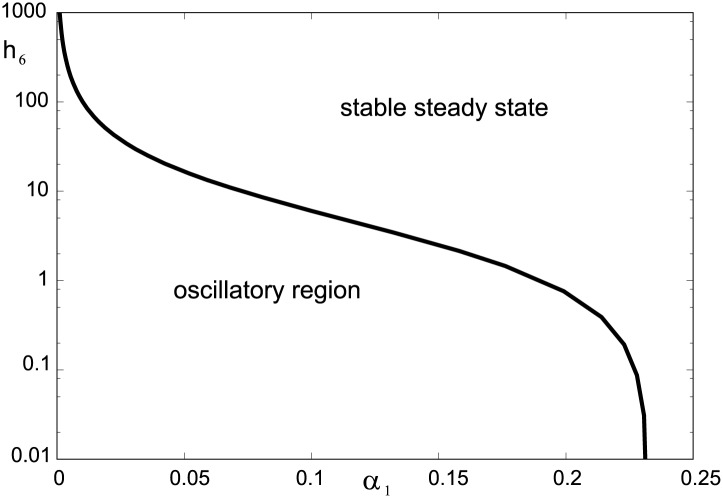
Bifurcation diagram in *α*_1_–*h*_6_ plane. Analytically determined Hopf bifurcation curve delineates the plane of the convex parameters into region of oscillations and region of stable steady states.

The formulae expressing the coefficients *c*_*j*_ in terms of the convex parameters are readily available by using symbolic manipulation software. As an example, here we use Condition (30) for a specific choice of parameters. As in our previous setting, let *h*_1_ = *h*_2_ = *h*_3_ = 10, *h*_4_ = *h*_5_ = 1 and *α*_2_ = *α*_3_ = 1; the two free parameters are *α*_1_ and *h*_6_. Fig 7 shows an analytically determined Hopf bifurcation curve (see Eq (53) in Appendix). Oscillations bifurcate from a stable steady state as *α*_1_ passes from the right to the left through the critical value *α*_1,*c*_ corresponding to the curve. The oscillations vanish via an infinite period as *α*_1_ approaches zero, where only bistability can occur. Variation of *h*_6_ corresponds to variation of A_*tot*_ due to Eq (27) and variation of *α*_1_ corresponds to variation of *k*_6_ and *k*_7_ according to Eq (20).

Emergence of oscillations can be interpreted in agreement with our numerical analysis as follows: when *α*_1_ > *α*_1,*c*_ the unstable subnetwork E_2_ + E_3_ is stabilized by strong coupling to the equilibrium subnetwork E_1_ but destabilizes the steady state below the critical value of *α*_1_. It is also a straightforward implication of Eq (53) that if *α*_1_ = 0, the Routh-Hurwitz criterion is never satisfied, which bears out earlier findings about minimal bistable system formed solely by Stage 2.

## Discussion

We have carried out analysis leading toward detailed understanding of emerging oscillatory dynamics in the widely used Huang-Ferrell model of the MAPK signalling, which is based on detailed mass action kinetics. The methods of stoichiometric network theory and classification used in this kind of analysis have been previously applied mostly to inorganic chemical oscillators, but we endeavored to demonstrate their usefulness in the context of biochemical networks. The advantage of compactness of the approach using kinetic rate expressions based on quasi-steady state assumptions, such as saturation kinetic terms, is replaced by readily performed stability analysis when the kinetics of elementary (or quasi-elementary) steps are retained regardless of the size of network. Systematic tools are available to find dynamically relevant subnetworks, determine the role of species in the dynamic modes resulting from complex competition of positive and negative feedback and, particularly, to help understand the nature of oscillatory modes [40, 41].

The Huang-Ferrell model has an in-built feature of taking into account distributive and avoiding processive phosphorylation. Using the symbols in this work, a processive sequence would be step 1 followed by an on site second phosphorylation forming A*B_1_ and subsequently B_2_ thus bypassing the steps 2 and 3. It is known that processive phosphorylation cannot have multiple steady states [46]. By the absence of the processive step, our reduction from the original mechanism shown in Table 2 to that in Table 3 preserves the dynamic instabilities despite neglecting reverse reaction steps. When going back from the reduced model to the original one, the hysteretic and oscillatory instabilities are preserved. In particular, the delumped complex A*B_1_ plays a role similar as B_2_, that is, of type Z species. Also, given the values of convex parameters, the coupling of reverse reactions may be indifferent to an instability or suppress it to a variable degree. Recently, a detailed model has been proposed by Rubinstein et al. [47] that combines both processive and distributive phosphorylation and takes into account an ordered process, whereby two different sites (tyrosine and threonine) are used sequentially for the first and second phosphorylation as well as dephosphorylation. They report oscillations of various types in an isolated Stage 2. A preliminary network analysis using our tools indicates several unstable subnetworks with topologies involving either processive or distributive steps, or unique combinations thereof, capable of generating oscillatory instability consistent with their findings. The specific roles of species elucidating interactions leading to oscillatory dynamics will be determined in our future work.

When comparing results of this work with earlier analysis of stability and oscillations in the models of MAPK cascades [8, 11, 15] we would like to point out that in many ways the results are consistent, but looked upon from different viewpoints. In particular, the conditions for bistability in a single MAPK tier assuming distributive phosphorylation are described by Markevich et al. [15] in terms of Michaelis-Menten (polynomial fractions) rate expressions with the conclusions that bistability is caused by several inhibitory loops: inhibition of the second phosphorylation by B, inhibition of the second dephosphorylation by B_2_, inhibition of the first phosphorylation and first dephosporylation by B_1_ and inhibition of both dephosphorylation steps by B. Of special importance is the competitive inhibition by the monophosphorylated form B_1_. These inhibitory effects were introduced in the model and shown to provide bistable switches. Oscillatory dynamics was then obtained by additional negative feedback loop from the double phosphorylated product to the top-tier kinase in a three-stage cascade [8] and later in a two-stage cascade or layers of such cascades [11]. An important aspect of using the mass action network is that no ad hoc inhibitory loops are needed, all the necessary negative feedbacks are in-built provided that suitable enzyme-substrate complexes are kept in the network as independent dynamical variables along with at least one of the enzymes (kinase A* or phosphatase C). We chose the kinase to be dynamical variable but the mass action network of a single stage is symmetric with respect to both enzymes so their roles could be reversed to observe bistability.

However, unlike phosphatase, the activated kinase cannot be fixed when the oscillatory dynamics is to take place within the Huang-Ferrell model. As indicated in numerical calculations by Qiao et al. [17], in the mass action model Stage I should be added for oscillations. Here we have examined this aspect rigorously and pointed out that activation/deactivation of A naturally takes part in oscillatory negative feedback without the need of assuming feedback on A* by B_2_. Also, we have shown that a crucial feature of the network resulting in positive feedback is competitive autocatalysis, which in essence is formed by competition of the substrates B and B_1_ for the activated kinase A* with the additional recycling pathway from B_2_ to B_1_ to B. In the oscillatory mode B_2_ and the complex A*B serve as temporary buffers, which implies a separation of the steps into fast and slow, in particular, the first phosphorylation cycle ratio *k*_2_/*k*_5_ is less than the ratio *k*_3_/*k*_4_ for the second cycle. These observations are mostly consistent with previous work based on the Michaelis-Menten rate expressions but go further by classifying the species and elucidating their role in oscillations simply by analyzing and ultimately reducing the detailed model rather than starting with the condensed Michaelian kinetic form and rationalizing its extensions.

Biological significance of the oscillatory mode in MAPK cascades is yet to be understood. Nonetheless, with the growing body of experimental evidence of oscillations in MAPK signalling, the conditions for occurrence of periodic dynamics in various models of MAPK signalling need to be checked and explained. Using reaction networks theory as presented in this work has the advantage of having systematic tools for explaining the oscillations as being caused by a core subnetwork possessing a proper combination of positive and negative feedback. Such subnetwork is rather straightforward to identify within possibly much larger network, provided that mass action/power law kinetics apply.

As a final remark, let us note that the stoichiometric constraints embodied in the collection of elementary subnetworks E_j_ can be conveniently used in determining a subset of the rate coefficients and/or steady state values provided that another subset is known from measurements or otherwise by using linear optimization targeted at the Hopf oscillatory instability applied to the convex cone. Our recent results provided unknown rate coefficients in the mechanism of catalase-glucose-oxidase oscillator that yield dynamics consistent with the observed oscillations and hold promise for other enzyme systems such as the MAPK.

## Appendix

### Part1—Equations for kinetic parametrization

Steady states of Eqs (12)–(17) together with the conservation Eqs (18) and (19) are given by the following expressions. There are three independent solutions $x_{j}^{\left(m\right)},\;m\in \left[\left(i\right),\left(ii\right),\left(iii\right)\right],j=1,\cdots ,6]$,

$$\begin{array}{ccc} x_{1}^{\left(m\right)} & = & k_{6}x_{6}^{\left(m\right)}/k_{7}, \end{array}$$ (31)

$$\begin{array}{ccc} x_{2}^{\left(m\right)} & = & \frac{z_{5}{\left[x_{6}^{\left(m\right)}\right]}^{2}}{z_{6}}+\frac{z_{7}x_{6}^{\left(m\right)}}{k_{4}k_{5}k_{7}}-{\text{A}}_{\text{tot}}\left(\frac{k_{2}}{k_{5}}+1\right)+{\text{B}}_{\text{tot}}, \end{array}$$ (32)

$$\begin{array}{ccc} x_{3}^{\left(m\right)} & = & \left({\text{A}}_{\text{tot}}k_{2}-k_{2}x_{6}^{\left(m\right)}\left(k_{6}/k_{7}+1\right)\right)/k_{5}, \end{array}$$ (33)

$$\begin{array}{ccc} x_{4}^{\left(m\right)} & = & {\text{A}}_{\text{tot}}-\left(k_{6}/k_{7}+1\right)x_{6}^{\left(m\right)}, \end{array}$$ (34)

$$\begin{array}{ccc} x_{5}^{\left(m\right)} & = & -\left(z_{5}{\left[x_{6}^{\left(m\right)}\right]}^{2}-{\text{A}}_{\text{tot}}k_{2}k_{3}k_{6}k_{7}x_{6}^{\left(m\right)}\right)/z_{6}, \end{array}$$ (35)

$$\begin{array}{ccc} x_{6}^{\left(i\right)} & = & -b_{4}, \end{array}$$ (36)

$$\begin{array}{ccc} x_{6}^{\left(ii\right)} & = & -(b_{3}+b_{2}), \end{array}$$ (37)

$$\begin{array}{ccc} x_{6}^{\left(iii\right)} & = & -(b_{3}-b_{2}), \end{array}$$ (38)

where the coefficients *b*_*j*_ are defined as

$$\begin{array}{ccc} b_{1} & = & \{{\left[{\left(\frac{3}{2}a_{1}a_{2}-a_{2}^{3}+a_{3}\right)}^{2}+{\left(a_{1}-a_{2}^{2}\right)}^{3}\right]}^{\frac{1}{2}}\\ & & -a_{2}^{3}+\frac{3}{2}a_{1}a_{2}+a_{3}{\}}^{\frac{1}{3}}, \end{array}$$ (39)

$$\begin{array}{ccc} b_{2} & = & i\left(b_{1}+\left(a_{1}-a_{2}^{2}\right)/b_{1}\right)\sqrt{3}/2, \end{array}$$ (40)

$$\begin{array}{ccc} b_{3} & = & a_{2}+b_{1}/2-\left(a_{1}-a_{2}^{2}\right)/\left(2b_{1}\right), \end{array}$$ (41)

$$\begin{array}{ccc} b_{4} & = & a_{2}-b_{1}+\left(a_{1}-a_{2}^{2}\right)/b_{1}. \end{array}$$ (42)

Here *b*_2_ contains the imaginary unit $i=\sqrt{-1}$ and the coefficients *a*_*j*_ read

$$\begin{array}{ccc} a_{1} & = & z_{3}/\left(3z_{2}\right), \end{array}$$ (43)

$$\begin{array}{ccc} a_{2} & = & z_{1}/\left(3z_{2}\right), \end{array}$$ (44)

$$\begin{array}{ccc} a_{3} & = & z_{4}/\left(2z_{2}\right), \end{array}$$ (45)

with *z*_*j*_ being functions of the kinetic parameters:

$$\begin{array}{ccc} z_{1} & = & k_{1}k_{2}k_{4}k_{6}k_{7}^{2}+k_{1}k_{2}k_{4}k_{6}^{2}k_{7}+k_{1}k_{4}k_{5}k_{6}k_{7}^{2}+\\ & & k_{1}k_{4}k_{5}k_{6}^{2}k_{7}-{\text{A}}_{\text{tot}}k_{1}k_{2}k_{3}k_{6}^{2}k_{7}, \end{array}$$ (46)

$$\begin{array}{ccc} z_{2} & = & k_{1}k_{2}k_{3}k_{6}^{3}+k_{1}k_{2}k_{3}k_{7}k_{6}^{2}, \end{array}$$ (47)

$$\begin{array}{ccc} z_{3} & = & k_{2}k_{4}k_{5}k_{7}^{3}+k_{2}k_{4}k_{5}k_{6}k_{7}^{2}-{\text{A}}_{\text{tot}}k_{1}k_{2}k_{4}k_{6}k_{7}^{2}-\\ & & {\text{A}}_{\text{tot}}k_{1}k_{4}k_{5}k_{6}k_{7}^{2}+{\text{B}}_{\text{tot}}k_{1}k_{4}k_{5}k_{6}k_{7}^{2}, \end{array}$$ (48)

$$\begin{array}{ccc} z_{4} & = & {\text{A}}_{\text{tot}}k_{2}k_{4}k_{5}k_{7}^{3}, \end{array}$$ (49)

$$\begin{array}{ccc} z_{5} & = & k_{2}k_{3}k_{6}\left(k_{6}+k_{7}\right), \end{array}$$ (50)

$$\begin{array}{ccc} z_{6} & = & k_{4}k_{5}k_{7}^{2}, \end{array}$$ (51)

$$\begin{array}{ccc} z_{7} & = & k_{2}k_{4}k_{6}+k_{2}k_{4}k_{7}+k_{4}k_{5}k_{6}+k_{4}k_{5}k_{7}-\\ & & {\text{A}}_{\text{tot}}k_{2}k_{3}k_{6}. \end{array}$$ (52)

### Part2—Hopf bifurcation condition in convex parametrization

The equality part of the Condition (30) for a specific choice of parameters *h*_1_ = *h*_2_ = *h*_3_ = 10, *h*_4_ = *h*_5_ = 1 and *α*_2_ = *α*_3_ = 1 reads

$$\begin{array}{c} \frac{83109575}{648}\alpha_{1,c}+\frac{8127230}{243}\alpha_{1,c}h_{6}+\frac{38535875}{216}\alpha_{1,c}^{2}+\frac{74379835}{1296}\alpha_{1,c}^{2}h_{6}+\frac{334417}{81}\alpha_{1,c}^{2}h_{6}^{2}+\\ \frac{1923125}{36}\alpha_{1,c}^{3}+\frac{809375}{36}\alpha_{1,c}^{3}h_{6}+\frac{1300475}{432}\alpha_{1,c}^{3}h_{6}^{2}+\frac{3500}{27}\alpha_{1,c}^{3}h_{6}^{3}-\frac{9738025}{243}=0\;. \end{array}$$ (53)

The Hopf bifurcation from a stable steady state occurs for a given *h*_6_ as *α*_1_ passes from right to left through the critical value *α*_1,*c*_.
